# Dysregulated but not decreased salience network activity in schizophrenia

**DOI:** 10.3389/fnhum.2013.00065

**Published:** 2013-03-07

**Authors:** Thomas P. White, James Gilleen, Sukhwinder S. Shergill

**Affiliations:** Department of Psychosis Studies, Institute of Psychiatry, King's College London London, UK

**Keywords:** schizophrenia, salience, cortical networks, fMRI, reward

## Abstract

Effective estimation of the salience of environmental stimuli underlies adaptive behavior, while related aberrance is believed to undermine rational thought processes in schizophrenia. A network including bilateral frontoinsular cortex (FIC) and dorsal anterior cingulate cortex (dACC) has been observed to respond to salient stimuli using functional magnetic resonance imaging (fMRI). To test the hypothesis that activity in this salience network (SN) is less discriminately modulated by contextually-relevant stimuli in schizophrenia than in healthy individuals, fMRI data were collected in 20 individuals with schizophrenia and 13 matched controls during performance of a modified monetary incentive delay (MID) task. After quantitatively identifying spatial components representative of the FIC and dACC features of the SN, two principal analyses were conducted. In the first, modulation of SN activity by salience was assessed by measuring response to trial outcome. First-level general linear models were applied to individual-specific time-courses of SN activity identified using spatial independent component analysis (ICA). This analysis revealed a significant salience-by-performance-by-group interaction on the best-fit FIC component's activity at trial outcome, whereby healthy individuals but not individuals with schizophrenia exhibited greater distinction between the response to hits and misses in high salience trials than in low salience trials. The second analysis aimed to ascertain whether SN component amplitude differed between the study groups over the duration of the experiment. Independent-samples *T*-tests on back-projected, percent-signal-change scaled SN component images importantly showed that the groups did not differ in the overall amplitude of SN expression over the entire dataset. These findings of dysregulated but not decreased SN activity in schizophrenia provide physiological support for mechanistic conceptual frameworks of delusional thought formation.

## Introduction

The finite capacity of our attentional and behavioral resources necessitates that we assign preferential salience to certain environmental stimuli, while limiting responses to others. Appropriately selecting the stimuli to which we assign salience is therefore a key component of adaptive behavior. Relatedly, the allocation of incentive salience to both primary and more abstract rewarding stimuli potently modulates behavior (Robbins and Everitt, 1996).

Electrophysiological recordings from the macaque striatum show that phasic dopaminergic responses to rewarding stimuli temporally mimic the prediction error of reward-value models, implicating this region as the source of a reinforcement signal required to adjust the probability of subsequent action selection (Schultz et al., 1997). Comparable human blood oxygenation-level dependent (BOLD) responses in striatum to novel, aversive and intense stimuli suggest that this response indexes salience-related features such as familiarity, contextual relevance and predictability more generally (Zink et al., 2004). A complementary model of the role of phasic striatal dopamine therefore proposes that the basal ganglia concertedly act as a centralized selection device, allocating attentional resources between competing motor programs in a contextually-relevant manner (Redgrave et al., 1999). The reward findings uphold this model insofar as test animals are generally required to shift attention to rewarding stimuli and carry out a motor program to realize their consumption.

Human BOLD findings also imply an analogous, attention-switching function in a cortical network comprising dorsal anterior cingulate cortex (dACC) and bilateral frontoinsular cortex (FIC), subsuming anterior insula (AI), and inferior frontal gyrus (IFG). These regions are consistently coactive with task-specific regions when stimuli are modulated in terms of cognitive, emotional and homeostatic salience, which implies they fundamentally code salience (Menon, 2011); they also exhibit a temporal signature dissociable from task-specific regions via seed-region correlation and independent component analysis (ICA) suggesting that they represent a salience network (SN; Seeley et al., 2007), Moreover, analyzing Granger-causal relationships from multi-task functional magnetic resonance imaging (fMRI) data, Sridharan et al. (2008) observed that right FIC activity consistently preceded and predicted activity in regions of the default mode network (DMN; medial prefrontal cortex, precuneus and bilateral angular gyrus), where activity is typically greater during times of introspection, and also regions of the central executive network (CEN; bilateral dorsolateral PFC and posterior parietal cortex), where activity is typically greater when attention is focused on environmental stimuli. They suggested that right FIC was therefore crucial in switching between these two contrasting modes of brain function.

The cortical SN appears to be a focus of pathology in schizophrenia. Structurally, these regions are amongst the most consistently observed sites of gray-matter reduction in the disorder (Ellison-Wright et al., 2008), and focal alterations in SN volume are observed to be associated with the severity of reality distortion in schizophrenia (Palaniyappan et al., 2011). Reduced functional connectivity has also been observed within the SN in schizophrenia compared to controls during volitional eye saccades (Tu et al., 2010) and at rest (Tu et al., 2012). However, within-SN dysconnectivity is not unequivocally apparent in schizophrenia. Resting-state connectivity between a FIC seed and other SN constituents was recently reported to be unaffected by schizophrenia, despite significant connectivity reductions in both DMN and CEN (Woodward et al., 2011). Combined assessment of within- and between-network connectivity in schizophrenia as compared with controls has revealed less consistent functional relationships within the SN and between the SN and DMN during passive perceptual stimulation (White et al., 2010a).

The monetary incentive delay (MID) task presents an adaptable framework for assessing SN functional modulation in schizophrenia. Investigating gains (but not losses) Walter et al. (2009, 2010) demonstrated that activity in dACC and a region encompassing AI and ventrolateral prefrontal cortex is more sensitively modulated at outcome in healthy individuals than schizophrenia patients in tasks that vary reward magnitude and those that vary reward probability. Furthermore, Waltz et al. (2010) reported significant group-by-outcome interactions in right FIC and pregenual ACC BOLD responses in a MID task involving both gains and losses, with controls exhibiting greater activity for gains than losses and patients exhibiting greater responses for losses than gains. While these latter findings are in line with anhedonic symptoms of schizophrenia, from a biological perspective salience should be attributed to both positive and negative events—potential rewards and dangers must both be appropriately detected. As a result, salience coding should be expectedly heightened to both positive and negative extremes. Moreover, the success with which potential losses and gains are respectively obtained or avoided should additionally contribute to salience coding to maximally adapt subsequent behavioral output.

The notion that individuals with schizophrenia exhibit not just muted salience attribution to conventionally salient stimuli but also aberrantly excessive salience attribution at other times is central to dominant theories of delusion formation (Kapur, 2003; Kapur et al., 2005). This suggests that attenuated reward signals in cortical SN and striatum (for review, see Heinz and Schlagenhauf, 2010) paint an incomplete picture. Here, we use spatial ICA of fMRI data to identify the cortical SN in individuals with schizophrenia and matched controls during performance of a modified MID task. We present analyses conducted to assess the explicit hypotheses that: (1) cortical SN activity focused in both dACC and FIC will be modulated by the salience of rewarding monetary stimuli at reward outcome in healthy individuals; (2) SN modulation by task and performance will be diminished in schizophrenia; and (3) despite this putative dysregulation in schizophrenia, the cortical SN will be no less evident in these individuals than in healthy controls over the duration of the task.

## Material and methods

### Participants

Twenty individuals satisfying DSM-IV criteria for schizophrenia (American Psychiatric Association, 1994) and 13 healthy controls were recruited to take part in the study. All participants were right-handed and groups did not differ significantly in terms of age or intelligence quotient (IQ) assessed using the National Adult Reading Test (NART; Nelson, 1982). Summary demographic and psychiatric-symptom details are provided in Table 1.

**Table 1:** **Sample details**.

**Measure**	**Group**	
	**Schizophrenia group (*n* = 20)**	**Healthy group (*n* = 13)**
**(A) GROUP MEAN DEMOGRAPHIC DETAILS. BRACKETED VALUES DENOTE STANDARD DEVIATION**		
Age (years)	36.9 (6.95)	31.3 (9.7)
Intelligence quotient (NART)	101.6 (11.62)	106.4 (9.1)
**(B) MEAN SCHIZOPHRENIA GROUP PANSS SCORES. BRACKETED VALUES DENOTE STANDARD DEVIATION**		
Positive	13.77 (6.92)	
Negative	13.31 (5.92)	
General	26.81 (6.5)	

NART, national adult reading test (Nelson, 1982); PANSS, positive and negative signs and symptoms of schizophrenia (Kay et al., 1987).

Diagnosis of schizophrenia was confirmed by assessment of clinical case notes and confirmation of suitability by each individual's consultant psychiatrist. Patients were recruited in a clinically stable condition and were excluded if presenting evidence of comorbid diagnosis or a medical disorder resulting in an IQ of less than 85. Symptom severity and classification were assessed using the Positive and Negative Syndrome Scale (PANSS; Kay et al., 1987). Sixteen patients were receiving treatment with atypical antipsychotic medications: olanzapine (*n* = 8); risperidone (*n* = 4); quetiapine (*n* = 2); clozapine (*n* = 1); and sulpiride (*n* = 1). The remaining four patients were receiving typical antipsychotic medications: zuclopenthixol (*n* = 2); flupentixol (*n* = 1); and chlorpromazine (*n* = 1). Chlorpromazine equivalent doses were computed for oral antipsychotic medications using data presented by Woods (2003). In the case of risperidone Consta injection, 25 mg Consta injection every 14 days was taken to equate to 4 mg oral risperidone per day, in accordance with the British National Formulary recommendation (Joint Formulary Committee, 2008). The average chlorpromazine-equivalent dose was 292.7 (range: 100–700) mg/day.

Healthy volunteers were recruited by local advertisement and excluded from study if: they reported a personal history of psychiatric or neurological illness or diagnosis of schizophrenia in a first-degree relative; they exhibited an IQ of less than 85; or they had a recent history of illicit substance use.

Ethical approval was provided by Essex 1 Research and Ethic Committee (08/H0301/116). All participants provided informed written consent and were given an inconvenience allowance for study participation plus additional payment proportional to task performance.

### Experimental procedure

**Figure 1:**
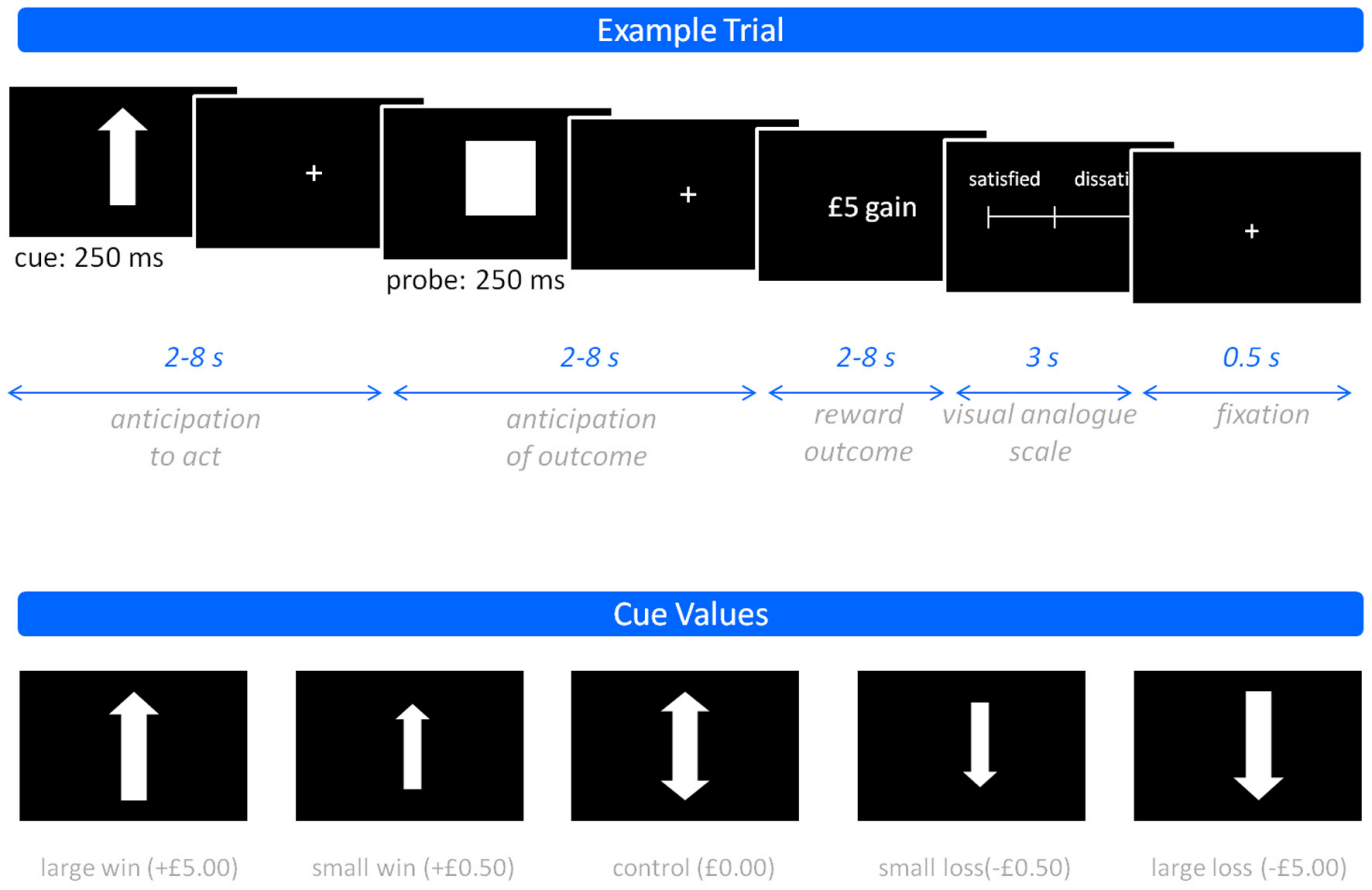
**The monetary incentive delay task.** Showing an example trial (top) and the various cue values used (bottom).

Participants performed a modified MID task (Knutson et al., 2001) comprising three 15-min sessions, each containing 48 trials split equally between the different experimental conditions. Participants viewed a screen, onto which visual stimuli were projected, using mirrors mounted on the scanner headcoil. Trials were categorized as either: win trials, in which pressing the button within a target time window resulted in the relevant reward (hit), while failure to do so (miss) resulted in no financial change; or loss trials, in which poor performance (miss) led to loss of the relevant monetary value, while good performance (hit) led to avoidance of this loss. Win and loss trials were further categorized according to the magnitude of their potential financial value (£5, £0.50, and £0). This permitted evaluation of graded incentive salience. As trials with a large potential reward/loss have greater financial implications, they should be considered more salient. Each trial began with a cue notifying trial type (win vs. loss; magnitude of reward), followed by a probe indicating when to perform the right index finger button press and then, following a delay, visual feedback indicating trial outcome. On completion of each trial, participants were required to manually report their feeling of subjective contentment using a visual analog scale (VAS), ranging from satisfied (1) to dissatisfied (9). A schematic representation of an example trial, including the timelines of phase 1 (anticipation to act), phase 2 (anticipation of outcome), and phase 3 (outcome), and the stimuli presented in the experiment is provided in Figure 1.

### MRI data acquisition

Four hundred and forty-eight gradient-echo echo-planar BOLD images (TR/TE: 2000/25 ms, flip angle: 75°, matrix: 64 × 64) were acquired on a 3 Tesla GE Excite II MR scanner (GE Healthcare, USA) during each run of the task. Each whole-brain image contained 38 non-contiguous slices of 2.4-mm thickness separated by a distance of 1 mm, and with in-plane isotropic voxel resolution of 3.4 mm.

### Behavioral data analysis

The time taken to make the button-press response following presentation of probe stimulus (reaction time; RT) was recorded for each trial. To reduce the effects of trials in which no response was made, the within-session median RT was calculated and these values averaged to give subject-specific mean RTs for each condition over the experiment as a whole. A repeated-measures ANOVA test was performed within SPSS (SPSS Inc., USA) to assess differences related to trial valence (win or loss), salience (£5 or £0.50), and group (healthy or schizophrenia). Similarly, the hit rate (percentage calculated on the basis of number of hits and total trials) was calculated for each condition averaged across sessions. Again, a repeated-measures ANOVA was conducted to assess differences relating to trial valence, salience and group. *Post-hoc T*-tests were performed to assess the specifics of significant main effects and between-factor interactions.

### fMRI data preprocessing and analysis

fMRI data were preprocessed using SPM5 (Wellcome Department of Imaging Neuroscience, University of London, UK). Data were realigned to the first image of the series, normalized to a standard-brain template and smoothed using an 8-mm FWHM Gaussian kernel.

Spatial ICA was performed on the pre-processed data using the Group ICA fMRI Toolbox (GIFT; http://icatb.sourceforge.net) within Matlab 7.8 (MathWorks, USA). GIFT uses a temporal concatenation approach during which data reduction is performed via multi-staged principal component analysis (PCA) and aggregation to generate common-group maps. These components are subsequently back-projected onto each individual's data to create subject-specific spatial maps with corresponding time-courses (Calhoun et al., 2008, 2009).

Prior to ICA, data dimensionality was estimated using Minimum Length Description criteria to be 64. Since model order determines network spatial characteristics including subnetwork parcellation, ICA was constrained to produce 64 components. ICA was performed using the Infomax algorithm (Bell and Sejnowski, 1995), and repeated five times with Icasso (Himberg et al., 2004) to maximize the stability of the derived components. Components were also scaled according to percent signal change to facilitate inter-subject comparisons of their time-courses. Back-reconstruction was carried out using GICA3 on the basis of previous empirical support for the accuracy of this method (Erhardt et al., 2011).

In light of previous observations that the dACC and FIC features of the SN are customarily dissociated into separate fMRI spatial components (Sridharan et al., 2008; White et al., 2010a), it was considered appropriate to attempt to identify these SN features independently. To this end, binary masks of (1) the dACC and (2) the FIC, were constructed from a downloadable SN map (http://findlab.stanford.edu/research; Shirer et al., 2012). Each binary mask was dilated by one voxel to favor components whose outside-mask loadings were greater in regions immediately proximate to the specified masks. Goodness-of-fit (GOF) was then assessed between each of these masks and the 64 whole-sample component maps. GOF was calculated by subtracting the mean *Z*-score of voxel values outside the mask from the mean *Z*-score of voxels within it (Greicius et al., 2004; Seeley et al., 2007) using Matlab 7.8 (MathWorks, USA). Components were ranked according to GOF and the best-fit component for each binary mask selected for subsequent investigation.

Having identified the two best-fit SN whole-sample components, their voxel-wise robustness was assessed statistically for the whole sample using one-sample *T*-tests conducted on whole-brain, back-reconstructed loading images for each participant. This identified the voxels with strongest loadings for these components, although it must be stressed that each component is a whole-brain component. To identify regions of strong positive loading, significance was ascribed according to a cluster-level criterion based on the spatial extent of suprathreshold voxel clusters. Voxel-level inclusion of *P* < 0.001 and cluster-level significance of *P* < 0.05 family-wise error corrected were used throughout this work. In addition, two-samples *T*-tests were performed to investigate between-group differences in the amplitude of expression of the SN components over the entire dataset. Little difference between individuals with schizophrenia and healthy controls was predicted here according to the hypothesis that SN activity is dysregulated rather than diminished.

To investigate modulation of SN activity by task-related events at outcome, first-level GLMs were applied to the back-projected time-courses of the two SN components, with the hypothesis that these events would predict activity less in individuals with schizophrenia than in healthy controls. This technique permits assessment of distributed, event-related brain activity and has advantages over the conventional voxel-wise, mass-univariate approach including: (1) it reduces the chances of Type-1 error inherent to mass-univariate analyses on account of the large number of tests involved in the latter; (2) it reduces the chances of Type-2 error likely in the latter as a consequence of attempts to stringently correct for these multiple tests; and (3) it presents a readily understandable summary statistic for a distributed feature of brain activity, which has been previously identified by virtue of its temporal congruity. The conjoined use of GLMs and ICA has for these reasons been successfully used in wide-ranging settings (for recent examples, see Caulo et al., 2011; Luckhoo et al., 2012; White et al., in press).

For the current GLM-ICA analyses we modeled the time-course of the BOLD response associated with the presentation of the visual stimuli throughout the task, by convolving a vector of delta functions for the onset and durations of these stimuli with the canonical haemodynamic response function. Regressors were included in the GLMs for events split by cue value, performance and phase. This resulted in 30 conditions (5 cue values × 2 performance outcomes × 3 phases) for each of the three sessions. When necessary, regressors were included for void trials during which no button-press response was registered. Session-specific realignment parameters were also included in the GLMs as covariates of no interest. Resulting, individual-specific GLMs were applied to the time-courses of the best-fit FIC and dACC components. Beta coefficients for responses at trial outcome were then exported into SPSS (SPSS Inc., USA) for statistical appraisal. Repeated-measures ANOVAs were carried out to assess the effects on the beta coefficients of within-subjects factors of reward salience (£5 or £0.50), performance (hit or miss) and trial valence (win or loss), and the between-subject factor of group (healthy or schizophrenia) for each of the best-fit components separately. *Post-hoc* confirmatory *T*-tests were performed to assess the direction of significant effects.

The relationship between psychiatric symptomatology and SN task modulation was assessed in the schizophrenia group using bivariate Pearson correlation between above-calculated beta coefficients and PANNS positive, negative and general psychopathology scores. Similarly, the relationship between antipsychotic medication and SN task modulation was investigated by assessing correlation between beta coefficients and chlorpromazine equivalent dosage in the same individuals. In a further analysis to investigate whether medication class predicted SN modulation, a repeated-measures ANOVA was conducted in the schizophrenia group to assess the effects on beta estimates of salience, performance and trial outcome as in the previous analyses; however, this analysis also included a binary covariate detailing whether each individual had been prescribed typical or atypical antipsychotic medication.

## Results

### Behavior

**Figure 2:**
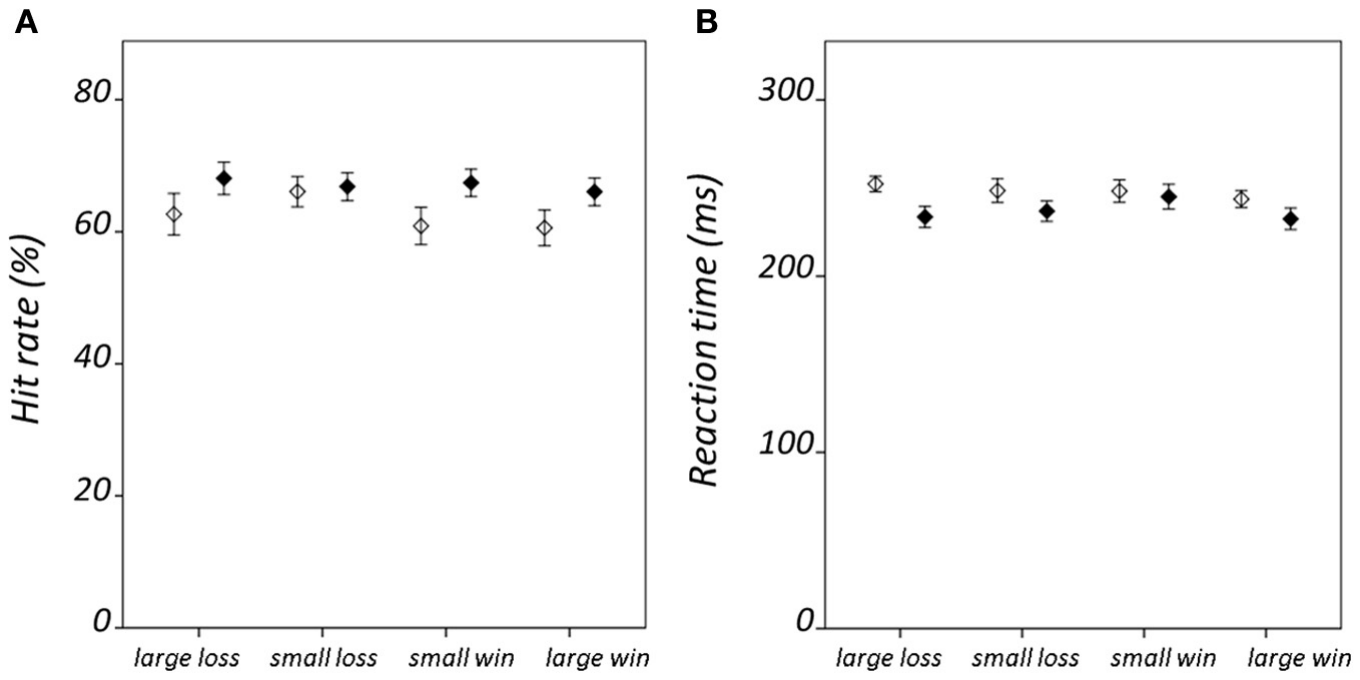
**Condition-specific behavioral results.** Showing **(A)** hit rate, and **(B)** reaction time. Error bars denote standard error of the mean. Healthy group results are shown in black and schizophrenia group results in white.

**Figure 3:**
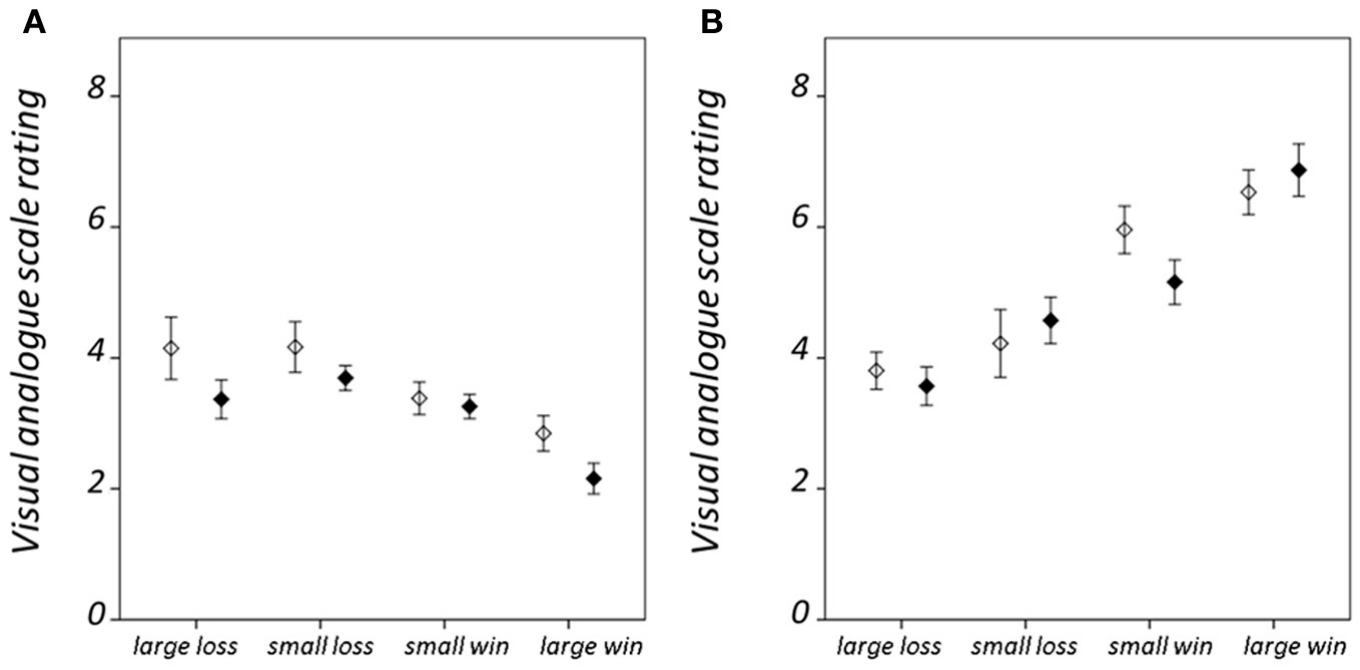
**Condition-specific visual analog scale (VAS) rating results.** For **(A)** hit trials, and **(B)** miss trials. VAS ratings range between 1 when the participant reported feeling “satisfied” and 9 when “dissatisfied.” Error bars denote standard error of the mean. Healthy group results are shown in black and schizophrenia group results in white.

Hit rate and RT results are summarized in Figure 2; statistical findings from the repeated-measures ANOVA conducted on these measures is provided in Table 2. There was a significant valence-by-salience interaction [*F*_(1, 32)_ = 9.02, *P* = 0.005] in RT. Subsequent *T*-tests demonstrated that while RT was significantly less for large win trials compared to small win trials [*T*_(32)_ = 3.76, *P* = 0.001], no significant difference was observed between large and small loss trials. A valence-by-group interaction [*F*_(1, 32)_ = 6.57, *P* = 0.015] was also observed in RT. Patients demonstrated significantly shorter RTs averaged across win trials (246.03 ± 24.07 ms) as compared to loss trials [250.42 ± 23.93 ms; *T*_(19)_ = 2.84, *P* = 0.011]. By contrast, healthy individuals exhibited non-significant differences in RT between these conditions [win trials: 238.82 ± 23.98 ms; loss trials: 235.26 ± 20.58 ms; *T*_(12)_ = 1.19, *P* = 0.255]. There was an additional weak trend toward a group effect in hit rate [*F*_(1, 32)_ = 2.78, *P* = 0.11]. This was observed on account of the increased number of void trials, for which no participant response was recorded, in the schizophrenia group as compared to the healthy group [healthy group: 1.33 ± 0.96 %; schizophrenia group: 5.75 ± 7.61 %; *T*_(32)_ = 2.07, *P* = 0.05]. VAS ratings categorized on the basis of trial type and group are presented in Figure 3. Main-effect and interaction statistics for VAS measures are shown in Table 3.

**Table 2:** **Modulation of hit rate and reaction time**.

**Measure**	**Effect**	***F*-value**	***P*-value**
Hit rate	V	1.49	0.231
	S	0.41	0.529
	G	2.78	0.105
	V × S	0.01	0.913
	V × G	0.66	0.423
	S × G	0.36	0.551
	V × S × G	1.54	0.224
Reaction time	V	0.72	0.790
	S	3.83	0.059
	G	1.97	0.171
	V × S	9.02	0.005
	V × G	6.57	0.015
	S × G	3.13	0.086
	V × S × G	0.26	0.874

Valence, V; Salience, S; Group, G.

**Table 3:** **Modulation of visual analog scale ratings**.

**Effect**	***F*-value**	***P*-value**
V	20.72	7 × 10^−5^
S	1.27	0.268
P	34.58	2 × 10^−6^
G	1.75	0.20
V × S	5.91	0.021
V × P	49.143	1 × 10^−6^
V × G	0.02	0.891
S × P	8.06	0.008
S × G	0.11	0.746
P × G	0.55	0.465
V × S × P	27.14	1 × 10^−4^
V × S × G	2.19	0.149
S × P × G	2.00	0.167
V × P × G	0.35	0.561
V × S × P × G	4.29	0.046

Valence, V; Salience, S; Performance, P; Group, G.

### SN identification and characterisation

**Figure 4:**
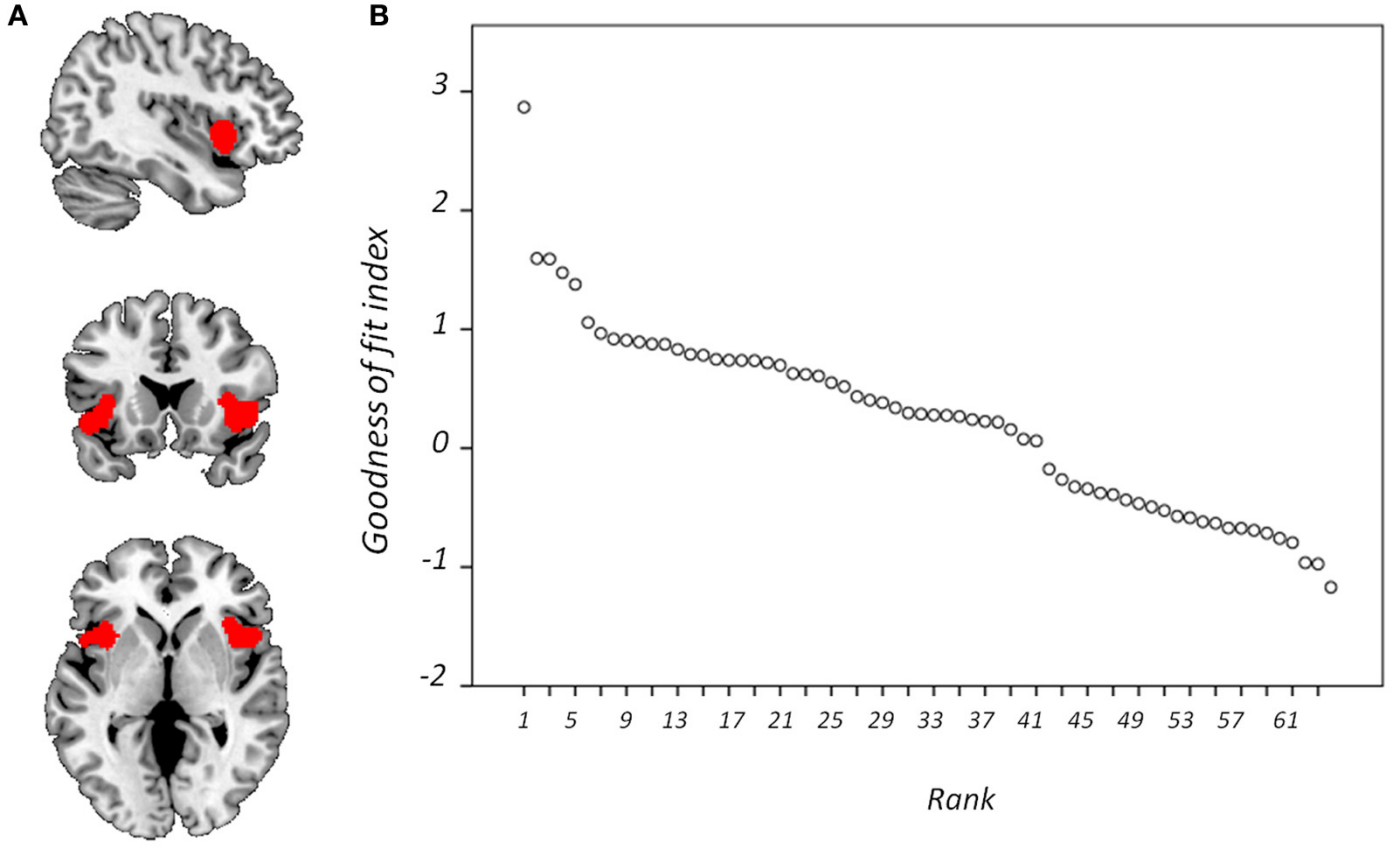
**Goodness-of-fit (GOF) with frontoinsular cortex mask.** Showing **(A)** the mask is overlaid onto a standardized T_1_-weighted image shown according to the neurological convention; and **(B)** GOF with this mask for each component displayed according to rank.

**Figure 5:**
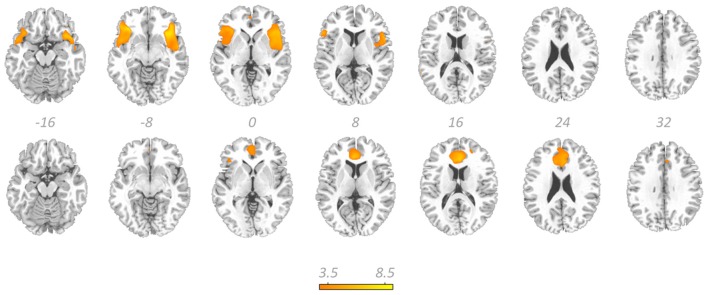
**The salience network.** Showing the best-fit frontoinsular cortex component (top row) and the best-fit anterior cingulate cortex component (bottom row) overlaid on a standardized T_1_-weighted image. Colorbar denotes *T*-scores from one sample *T*-tests and relates to both components. These images are thresholded at uncorrected *P*-threshold of 0.001.

**Figure 6:**
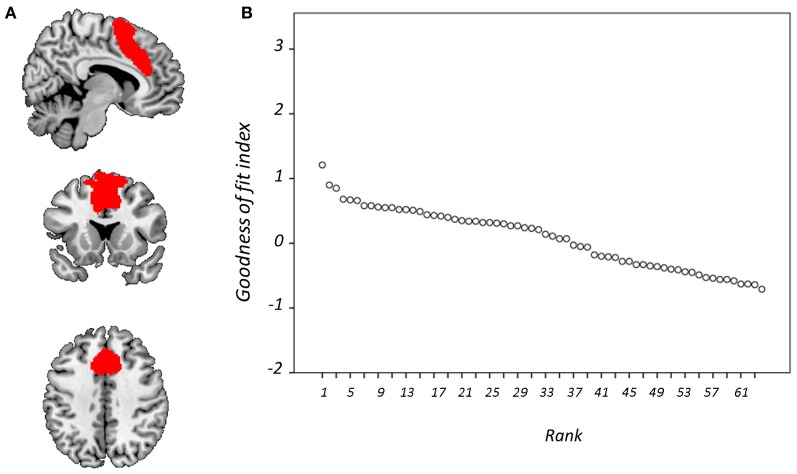
**Goodness-of-fit (GOF) with anterior cingulate cortex mask.** Showing **(A)** the mask is overlaid onto a standardized T_1_-weighted image shown according to the neurological convention; and **(B)** GOF with this mask for each component displayed according to rank.

Figure 4 displays GOF scores between the FIC mask and each of the 64 whole-sample components. The GOF score for the best-fit component was 2.87. This *Z*-score equates to a *P*-value of 0.004, and as such this component can be confidently declared to focus on FIC regions. A one-sample *T*-test on individual-specific component maps for the best-fit component demonstrated significant positive clusters in bilateral IFG and anterior insula and is displayed in Figure 5. Statistical characteristics of its gray-matter foci are presented in Table 4(A). Figure 6 displays GOF scores between the dACC mask and each of the 64 whole-sample components. The best-fit dACC component, whose whole-sample GOF with the dACC mask was 1.38 (which equates to a *P*-value of 0.168) and had maximal loadings in medial prefrontal regions. This component is displayed in Figure 5 and statistics relating to its gray-matter foci are presented in Table 4(B).

**Table 4:** **One-sample *T*-test results for the best-fit salience network components**.

**Brain structure (Brodmann area)**	**MNI coordinates**	***T*-value**	**Cluster size (k**_**E**_**)**
**(A) SIGNIFICANT GRAY-MATTER FOCI OF THE BEST-FIT FRONTO-INSULAR CORTEX COMPONENT**			
Left inferior frontal gyrus (47)	−34 18 −12	8.11	2641
Left insula (13)	−44 10 −2	5.81	2641
Left superior temporal gyrus (22)	−54 14 −6	5.81	2641
Right inferior frontal gyrus (47)	34 30 −10	5.86	2108
Right superior temporal gyrus (38)	46 10 −10	4.58	2108
Right anterior cingulate gyrus (32)	2 48 0	3.29	76
**(B) SIGNIFICANT GRAY-MATTER FOCI OF THE BEST-FIT ANTERIOR CINGULATE CORTEX COMPONENT**			
Left anterior cingulate gyrus (24)	−4 32 14	6.60	2053
Right anterior cingulate gyrus (24)	6 28 18	6.45	2053
Right anterior cingulate gyrus (32)	2 48 2	4.61	2053
Right inferior frontal gyrus (47)	46 26 4	4.09	91

### Between-group differences in SN component amplitude

Whole-brain examination of between-group amplitude differences in the best-fit FIC and dACC components conducted using two-samples *T*-tests revealed no significant clusters (using either an uncorrected or FWE-corrected cluster level of *P* < 0.05 and a voxel-level threshold of *P* < 0.001).

### SN activity at reward outcome

**Figure 7:**
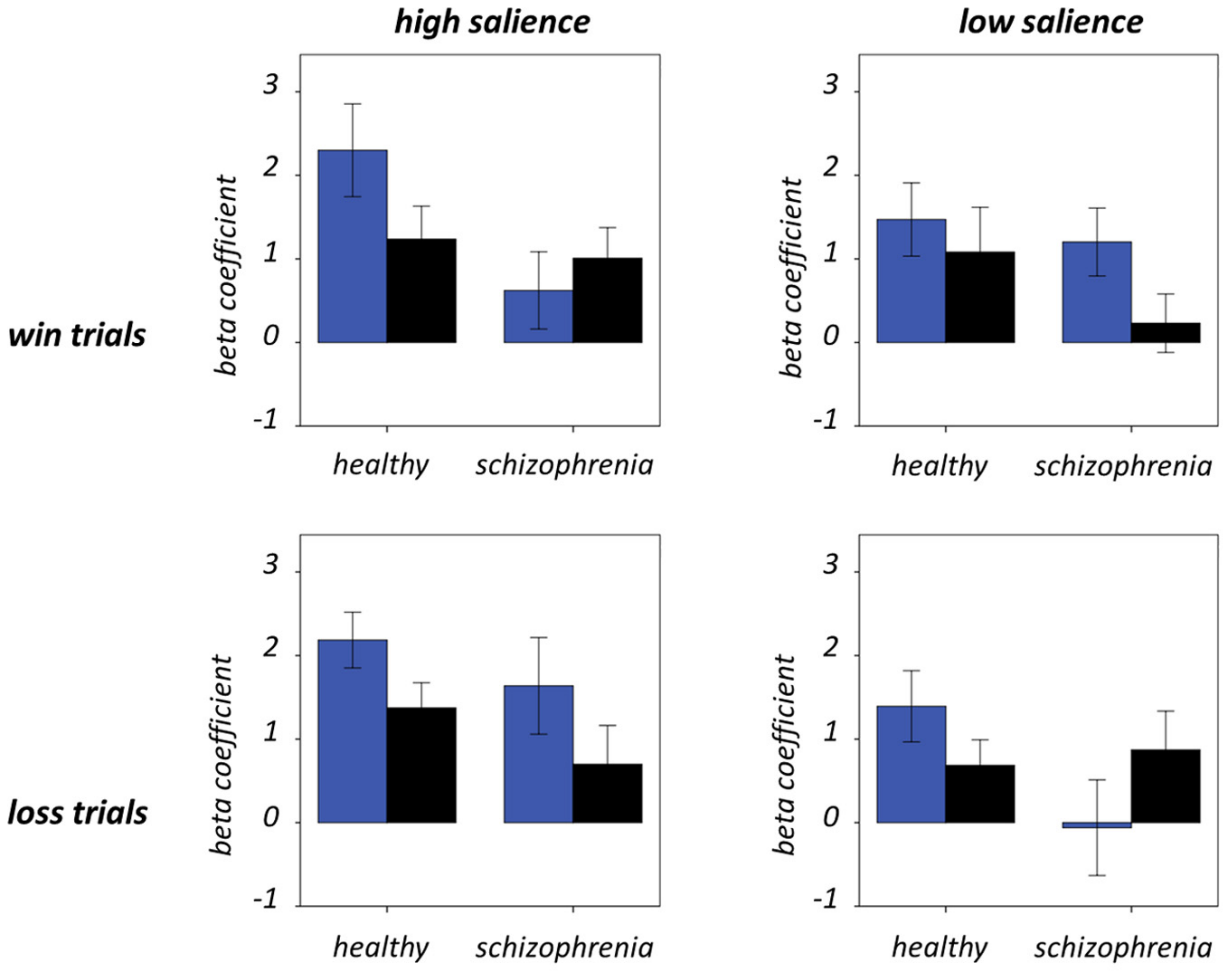
**Frontoinsular cortex component activity at trial outcome.** Condition-specific beta coefficients are shown, with blue and black bars denoting hit and miss trials, respectively. Error bars denote standard error of the mean.

**Figure 8:**
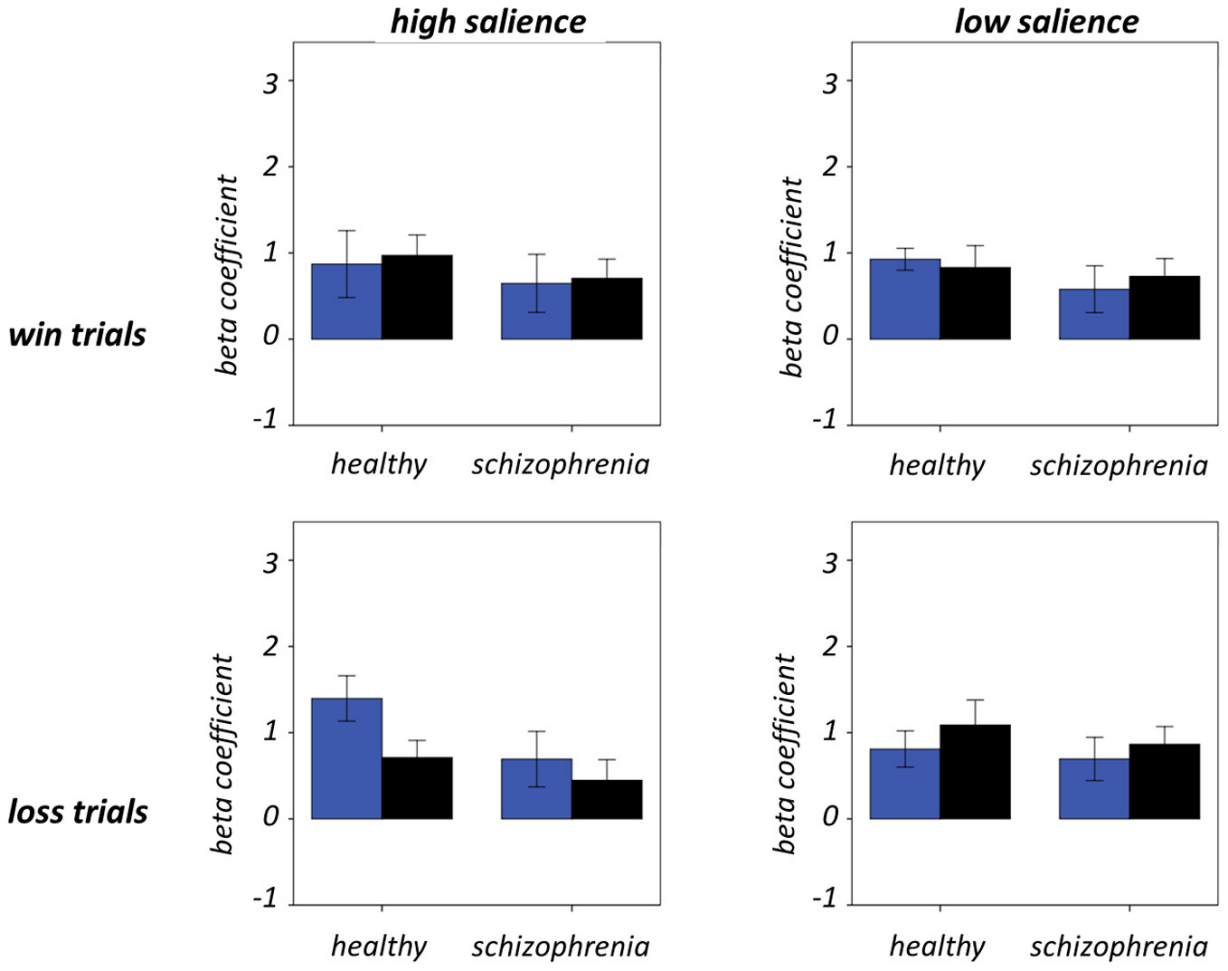
**Anterior cingulate cortex component activity at trial outcome.** Condition-specific beta coefficients are shown, with blue and black bars denoting hit and miss trials, respectively. Error bars denote standard error of the mean.

Figure 7 and Table 5 present beta coefficients for the best-fit FIC component at time of trial outcome. As is shown in Table 6, there was an overall main effect of group [*F*_(1, 32)_ = 4.82, *P* = 0.036]. Subsequent independent samples *T*-tests revealed that beta estimates for schizophrenia patients averaged over conditions were significantly smaller than those for healthy individuals. Interestingly, a significant salience-by-performance-by-group interaction was also observed [*F*_(1, 32)_ = 4.280, *P* = 0.047]. Healthy individuals displayed a trend toward greater responses for hits compared to misses (across both valences of conditions) in the high salience trials [*T*_(12)_ = 2.055, *P* = 0.061] and a non-significant effect for low salience trials [*T*_(12)_ = −1.730, *P* = 0.107]; by contrast, performance did not significantly modulate FIC response for either high or low salience trials in the schizophrenia group [high salience: *T*_(19)_ = 0.483, *P* = 0.634; low salience: *T*_(19)_ = −0.483, *P* = 0.966]. There was also a highly significant valence-by-salience interaction on FIC activity at reward outcome [*F*_(1, 32)_ = 11.353, *P* = 0.002]. A follow-up paired-samples *T*-test of the full study sample revealed that, while there was a non-significant difference in FIC modulation between high and low salience conditions for win trials [*T*_(32)_ = 0.944, *P* = 0.352], high salience loss trials evoked greater responses than corresponding low salience trials [*T*_(32)_ = 3.482, *P* = 0.001]. No other main effects or between-factor interactions were significant for this component. For the best-fit ACC component task modulation at reward outcome is shown in Figure 8, and beta estimates summarized in Table 5. No main effects or between-factor interactions were significant at conventional statistical thresholds, as is shown in Table 7.

**Table 5:** **Beta coefficients for salience network responses at outcome**.

**Condition**	**FIC component**		**ACC component**	
	**Healthy group**	**Schizophrenia group**	**Healthy group**	**Schizophrenia group**
Large win—hit	2.30 (2.07)	0.62 (2.07)	0.87 (1.45)	0.65 (1.51)
Large win—miss	1.24 (1.48)	1.01 (1.64)	0.97 (0.89)	0.71 (1.00)
Small win—hit	1.47 (1.64)	1.20 (1.81)	0.93 (0.47)	0.58 (1.21)
Small win—miss	1.08 (2.00)	0.23 (1.57)	0.83 (0.95)	0.73 (0.92)
Large win—hit	1.39 (1.59)	−0.06 (2.56)	0.81 (0.78)	0.69 (1.12)
Large win—miss	0.69 (1.14)	0.87 (2.06)	1.09 (1.09)	0.87 (0.92)
Large win—hit	2.19 (1.25)	1.64 (2.59)	1.40 (0.98)	0.69 (1.44)
Large win—miss	1.37 (1.12)	0.70 (2.07)	0.71 (0.74)	0.45 (1.07)

Group mean values and their standard deviation in brackets.

Frontoinsular cortex, FIC; Anterior cingulate cortex, ACC.

**Table 6:** **Modulation of frontoinsular cortex component activity by task**.

**Effect/interaction**	***F*-value**	***P*-value**
V	0.037	0.848
S	1.282	0.266
P	2.467	0.126
G	4.820	0.036
V × S	11.353	0.002
V × P	0.112	0.740
V × G	0.082	0.776
S × P	2.092	0.158
S × G	0.263	0.612
P × G	1.099	0.302
V × S × P	0.584	0.450
V × S × G	0.356	0.555
V × P × G	0.175	0.679
S × P × G	4.280	0.047
V × S × P × G	0.025	0.876

Valence, V; Salience, S; Performance, P; Group, G.

**Table 7:** **Modulation of anterior cingulate cortex component activity by task**.

**Effect/interaction**	***F*-value**	***P*-value**
V	0.188	0.667
S	0.176	0.678
P	0.073	0.789
G	1.299	0.263
V × S	0.008	0.929
V × P	0.797	0.379
V × G	0.130	0.721
S × P	3.37	0.077
S × G	0.537	0.469
P × G	0.290	0.594
V × S × P	3.247	0.081
V × S × G	0.527	0.473
V × P × G	0.027	0.869
S × P × G	1.055	0.312
V × S × P × G	0.131	0.720

Valence, V; Salience, S; Performance, P; Group, G.

### Relationships with symptom and medication

No statistically significant relationship was observed between SN task modulation and psychiatric symptomatology, medication dosage or medication class.

## Discussion

This salience-focused fMRI study of individuals with schizophrenia and matched control subjects employed spatial ICA to identify components of brain activity with maximal spatial correspondence with two principal features of the SN, namely the FIC and dACC; modulation of the activity of these components at trial outcome was then investigated to test the hypothesis that SN would be temporally dysregulated in schizophrenia.

The best-fit FIC group component exhibited a high GOF with the FIC mask (*Z* = 2.87, *P* = 0.004). It was characterized by maximal positive loadings in bilateral regions principally including AI and IFG, and can therefore be confidently described as exhibiting substantial FIC focus. Its hemispherically bilateral distribution is in accord with numerous fMRI ICA characterizations of the SN (for example, Seeley et al., 2007; Sridharan et al., 2008; White et al., 2010b), although it is noted that right FIC has been ascribed particular, influential roles in cognitive switching (Sridharan et al., 2008). GLM analysis of activity in this component at trial outcome produced findings of amplitude modulation suggestive of its role in salience coding.

A significant salience-by-performance-by-group interaction on FIC component activity was evident at trial outcome. In healthy individuals a trend toward greater responses for hits than misses was observed for high salience trials but less robustly for low salience trials. Since the outcome of a specific trial potently modulates its financial implications, and the differential consequences of the hit or miss are greater in high salience trials, it is unsurprising that the cortical system purported to encode salience exhibits modulation of activity in this manner in healthy individuals. An interesting addendum here is that FIC responses to hits exceed those to misses across high salience trials of both reward valences. Learning from a large-magnitude failure is an important aspect of adaptive behavior, and is contingent on importance being placed on the event in question; as such, it is somewhat surprising that this is not reflected in FIC activity at reward outcome.

Contrary to the healthy individuals, FIC component activity was not significantly modulated by performance as a function of salience in the individuals with schizophrenia. Non-significant performance effects were observed for both the high and low salience trial types. This demonstrates a functional impairment in FIC regulation in these individuals with potential behavioral repercussions. Efficient reinforcement learning must rely on determination of not only the potential salience of environmental events (as would be demonstrated by a main effect of salience) but also how this salience changes contextually (as would be demonstrated in this instance by a salience-by-performance interaction). That this effect is not evident in individuals with schizophrenia provides further physiological support for reinforcement learning deficits in these individuals (Evans et al., 2011; Maia and Frank, 2011).

There was also a significant salience-by-valence interaction on FIC activity at trial outcome. Across the whole sample, responses for large-loss hits exceeded those for small-loss hits, while the corresponding comparisons for win trials were insignificant. These findings provide a cortical correlate of “loss aversion,” by which individuals exhibit increased sensitivity for loss compared to gain (Tversky and Kahneman, 1979). Previous behavioral evidence suggests that this phenomenon is reduced in individuals with schizophrenia (Tremeau et al., 2008), representing a failure in the integration of affective and cognitive systems. Thus, while the current data highlight a finely-tuned aspect of SN function, it appears that the FIC does not act as the physiological substrate for the loss-aversion deficits previously observed in individuals with schizophrenia. Nevertheless, these findings of enhanced FIC sensitivity to negative events are broadly concordant with previous observations that right AI is influential in processing emotionally negative stimuli, such as those evoking feelings of disgust (Phillips et al., 1997).

The responses of the FIC component at trial outcome were consistently smaller in individuals with schizophrenia as compared to healthy individuals, as was demonstrated by the main effect of group in the repeated-measures ANOVA. This finding demonstrates that activity at this time was less tightly linked to the environmental stimuli in these individuals. While this is in itself noteworthy, the importance of this finding is magnified by the concomitant observations that contrasting features of FIC activity remain unchanged in the disorder. Despite the between-group differences in SN task modulation, voxel-wise comparison revealed no significant between-group difference in amplitude of SN expression over the dataset. This result suggests that the SN is similarly active in schizophrenia and that this network is similarly coherent in the disorder, thus acting as an integrated system (as reported by Woodward et al., 2011), despite exhibiting attenuated task-related responses in schizophrenia. In summary, these data suggest that the SN is dysregulated in schizophrenia rather than attenuated *per se*. This work, similarly to EEG reports of increased background oscillatory activity in the face of decreased event-related activity (Winterer et al., 2004), therefore provides evidence of a generalized failure to appropriately recruit task-relevant brain structures in schizophrenia. Unconventional recruitment of SN structures has particular clinical relevance in light of the putative consequences of this aberrance in the formation and shaping of thoughts. According to the aberrant salience hypothesis of psychotic illness (Kapur, 2003; Kapur et al., 2005), salience attribution is not only diminished for events to which salience is usually attributed, but also increased for events to which salience would not normally be assigned. This paper therefore adds a useful extension to the sizeable literature reporting diminished responses during reward (and more generally during salience processing) in regions including FIC and ventral striatum (for review, see Heinz and Schlagenhauf, 2010).

Our results relating to SN activity in ACC are less clear—no main effects or between-factor interactions were significant—but nevertheless raise important methodological issues related to the study of brain networks using ICA. The best-fit ACC component exhibited a GOF of 1.38 with the dACC mask. This value is at the 17th percentile and as such would not permit rejection of the null hypothesis (that the component does not significantly fit the mask) according to conventional statistical thresholds. Nevertheless, this component's global maximal positive loadings were in ACC and its significant positive clusters were limited to ACC and IFG. From this perspective, it can be validly adjudged a reasonable ACC component, albeit one whose ACC focus was more ventral than is conventional for the SN, given its extension into subgenual regions. The use of objective measures such as the GOF index (Greicius et al., 2004; Petrella et al., 2011) to identify components is preferential to visual inspection but is not without potential pitfall. Lability of distributed large-scale networks should be expected in view of variability of contemporaneous task demands and the psychological processes exercised. On these grounds, if activity in independent networks is coordinated over the course of an experiment they might reasonably be expected to be amalgamated into conglomerate spatial components. Despite the SN being initially identified by procedures such as ICA and seed-region connectivity (Seeley et al., 2007), this problem is particularly likely for networks such as the SN, which are responsible for processing stimuli at a fundamental level and hence likely to be coactive with task-specific regions in numerous tasks.

Antipsychotic medication is a potentially critical confounder of the study of reward salience systems in schizophrenia on account of its dopaminergic mode of action. However, several reports suggest that atypical antipsychotics have a normalizing effect on cerebral activity (for example, Lane et al., 2004). Confounding medication effects cannot be totally discounted in the present study; however, no significant relation was observed between chlorpromazine equivalent dosage or medication class and SN task modulation. While follow-up study in a drug-naïve sample is required to explicitly discount medication confounding effects, it is relevant that aberrant striatal activity during reward processing in schizophrenia has been shown to predate antipsychotic medication treatment (Schlagenhauf et al., 2009).

The application of GLMs to component time-courses lessens the multiple-comparison problem inherent in mass-univariate assessments of whole-brain activity using fMRI. However, a limitation of this procedure is that it does not permit fine-tuned regional inferences, since resultant beta coefficients relate to the component as a whole. As such, their utility depends on the justified selection of meaningful components of activity for GLM assessment. This criterion is met by the current analysis (in particular for the FIC component), given the spatial concordance of the current SN components with previous characterizations of the SN (Sridharan et al., 2008; Shirer et al., 2012).

A further limitation of this work is the employment of uneven sample sizes between the study groups. However, it is not considered that this difference significantly contributed to the findings presented. Since the healthy group sample was sufficient to detect wide-ranging within-group effects at conventional statistical thresholds, it can be reasonably claimed that the healthy group sample characteristics represent a realistic approximation of the population characteristics. Furthermore, our healthy sample was of comparable magnitude to those employed in previous fMRI investigations of abnormal reward processing in schizophrenia (for review, see Heinz and Schlagenhauf, 2010).

This work, in line with recent observations confirms the importance of FIC and to a lesser degree ACC in processing salient stimuli, and presents evidence of SN activity as a useful index of cognitive processes such as loss aversion. SN task modulation is more weakly apparent in schizophrenia as predicted by the considerable literatures of aberrant reward and target processing in the disorder (Kiehl and Liddle, 2001; Walter et al., 2009). The observation that the FIC component is not significantly reduced in amplitude in patients compared to healthy controls, when assessed in its entirety rather than from an event-related perspective, suggests that SN activity is not simply attenuated but rather temporally dysregulated in schizophrenia. This indirectly implies abnormally high SN activity at other times, in line with theories of abnormal salience attribution in the disorder.

## Funding

Funding was provided by the European Research Council ERC Consolidator Grant, #311686 and the NIHR Mental Health Biomedical Research Centre at the SLaM NHS Trust and King's College London, to SS.

### Conflict of interest statement

The authors declare that the research was conducted in the absence of any commercial or financial relationships that could be construed as a potential conflict of interest.
