# Corrigendum: Dysregulated but not decreased salience network activity in schizophrenia

**DOI:** 10.3389/fnhum.2019.00346

**Published:** 2019-10-09

**Authors:** Thomas P. White, James Gilleen, Sukhwinder S. Shergill

**Affiliations:** Department of Psychosis Studies, Institute of Psychiatry, King's College London, London, United Kingdom

**Keywords:** schizophrenia, salience, cortical networks, fMRI, reward

In the original article, we neglected to include the funder “European Research Council ERC Consolidator Grant #311686” and the “NIHR Mental Health Biomedical Research Centre at the SLaM NHS Trust and King's College London” to SS.

A Funding statement has therefore been added to the published article:

“Funding was provided by the European Research Council ERC Consolidator Grant, #311686 and the NIHR Mental Health Biomedical Research Centre at the SLaM NHS Trust and King's College London, to SS.”

The authors apologize for this error and state that this does not change the scientific conclusions of the article in any way. The original article has been updated.

